# Atypical bullous systemic lupus erythematosus with features of linear IgA bullous dermatosis

**DOI:** 10.1016/j.jdcr.2024.12.040

**Published:** 2025-02-07

**Authors:** Roy Jiang, Amanda G. Zhou, Christine J. Ko, Michael Girardi, Ailish Hanly, Catherine Xie, Ana Preda-Naumescu, Jeffrey R. Gehlhausen, Mary M. Tomayko

**Affiliations:** a Department of Dermatology, Yale School of Medicine, New Haven, Connecticut; b Department of Pathology, Yale School of Medicine, New Haven, Connecticut

**Keywords:** bullous SLE, immunobullous disorder, linear IgA dermatosis

## Introduction

Bullous systemic lupus erythematosus (BSLE) is a rare immunobullous disorder mediated by antibodies toward collagen VII in the setting of SLE, while linear IgA bullous dermatosis (LABD) is characterized by IgA deposition at the basement membrane as detected by direct immunofluorescence (DIF). While SLE may exist concomitantly with LABD, cases of BSLE with IgA only deposition on DIF are atypical. Here, we present the case of a pediatric patient provisionally diagnosed with LABD, who subsequently developed severe SLE symptoms including lupus nephritis and neurological sequelae. This case demonstrates the importance of reassessing a given diagnosis, especially as new symptoms and laboratory findings arise.

## Case report

**Fig 1:**
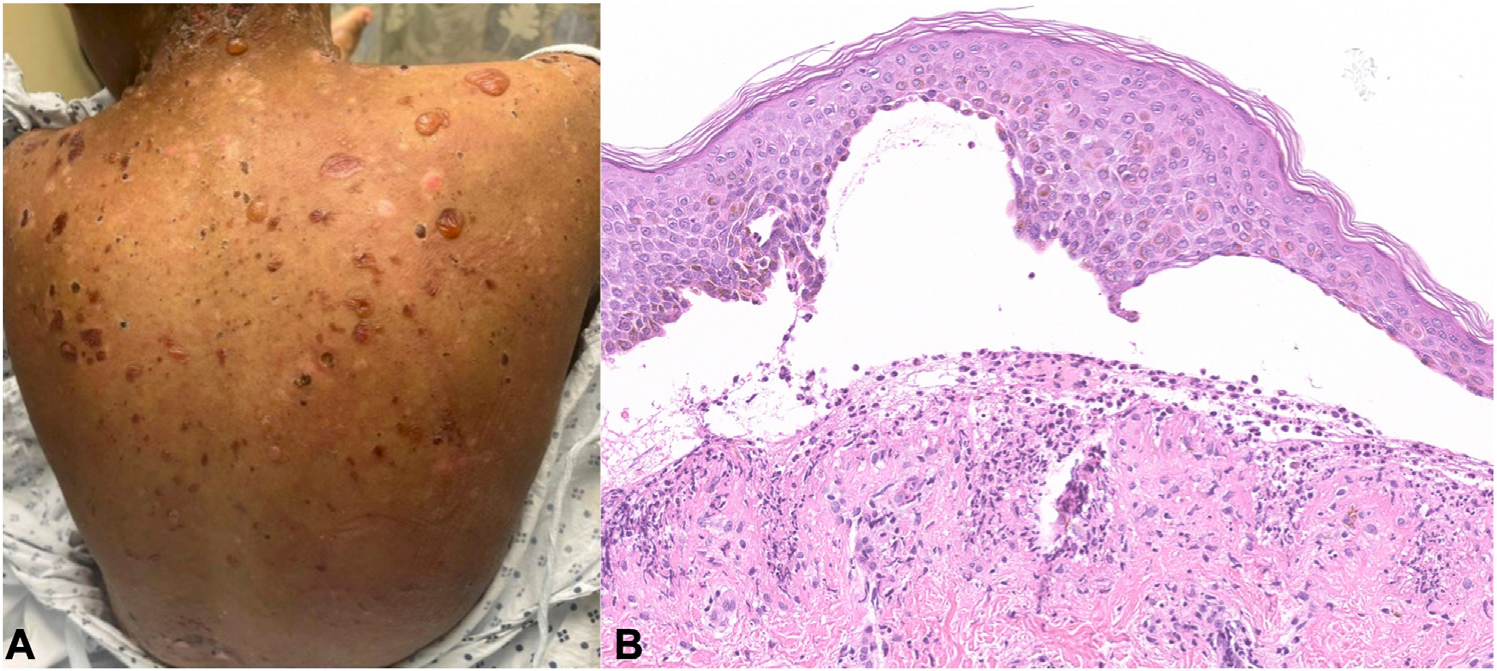
Initial presentation. (**A**) Exam findings on initial presentation of scattered vesicles and bullae on pink pruritic plaques over the back. (**B**) Initial H&E of skin biopsy sample showing subepidermal acantholysis and neutrophilic infiltrate at the DEJ. *DEJ*, Dermal-epidermal junction.

A 13-year-old Black female developed pruritic bullae that began over her lips and progressed over 2 months to involve her trunk and extremities (Fig 1, *A*). Concurrently, she developed urticarial papules/plaques. She was treated by urgent care and emergency medicine with a 6-day oral prednisone taper, amoxicillin/clavulanic acid, topical hydrocortisone 2.5%, and mupirocin 2% without improvement.

**Fig 2:**
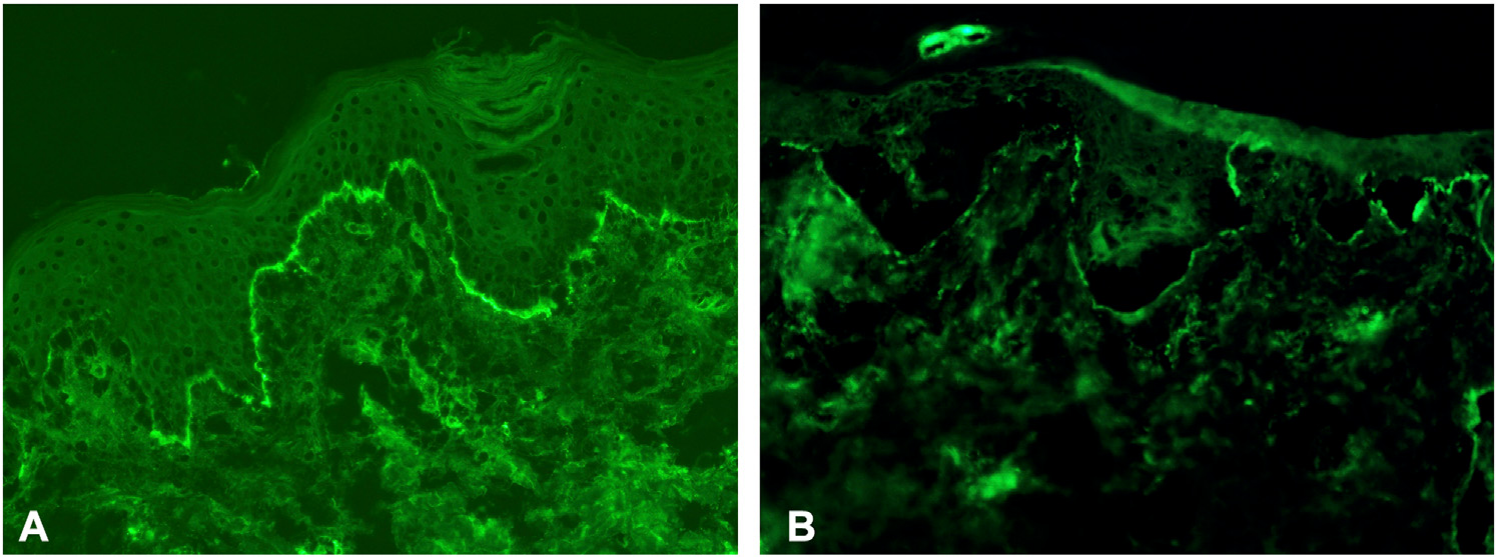
IgA immunofluorescence studies. (**A**) DIF showing linear IgA deposition along DEJ. (**B**) Dermal side IgA staining on DIF performed on SSS processed initial skin biopsy sample. *DEJ*, Dermal-epidermal junction; *DIF*, direct immunofluorescence; *SSS*, salt split skin.

On repeat presentation to the emergency department, she denied fever, arthralgia, or hematuria. Skin biopsies demonstrated a subepidermal split with dermal neutrophils (Fig 1, *B*). DIF showed linear deposition of IgA (without IgG or IgM) and C3 at the dermal-epidermal junction (Fig 2, *A*). U-serrations were observed, and dermal IgA deposition (without roof deposition) was seen on salt split skin (SSS) analysis (Fig 2, *B*). Initial labs demonstrated normocytic anemia (Hgb 8.3, range 11.4-14.7 g/dL). CRP (1.9 mg/L, range <10 mg/L), BP180 IgG (5 U/ml, range <14 U/ml) and BP230 IgG (<9 U/ml, range <9 U/mL) were within normal limits. Serum Desmoglein-1 (Dsg1) and −3 (Dsg3) IgGs were elevated at 30 U/ml (range <20 U/ml) and 80 U/ml (range <20 U/ml), respectively. The patient was started on prednisone 0.5 mg/kg/daily and given a single subcutaneous dose of dupilumab 600 mg. Oral dapsone was deferred given the normocytic anemia.

Three months after the onset of blisters and 5 weeks after her emergency department presentation, the patient was admitted for increasing skin pain, peripheral edema, and hypoalbuminemia, despite prednisone. She received a single dose of oral dapsone 25 mg (after negative G6PD deficiency testing) that was discontinued after she developed acute kidney injury with proteinuria. Kidney biopsy suggested lupus nephritis and therefore SLE; an ANA collected prior to admission was positive at 1:2560. Subsequent testing for other autoantibodies showed high serum SSA, SSB, Sm, and RNP autoantibodies (>240, 108, >310, > 240, U/ml, respectively), along with a low double-stranded DNA (13.0 U/ml) and positive rheumatoid factor (89.4 U/ml). Anticollagen VII IgG ELISA was negative.

Her course was complicated by ICU admission for seizures thought to be due to hypertension from lupus nephritis. She developed purple macules on her extremities consistent with cutaneous small vessel vasculitis. Transthoracic echocardiogram to rule out endocarditis was negative, and cryofibrinogen/cryoglobulin and ANCA screens were unremarkable. The patient was treated with 4 days of methylprednisolone, one dose of rituximab (1 g), and cyclophosphamide (750 mg/m^2^) before transitioning to mycophenolate (500 mg twice daily), hydroxychloroquine (300 mg daily), and prednisone (1.0 mg/kg daily), which stabilized her skin disease. Her prednisone was tapered to 5 mg daily and her skin remained clear 6 months later.

## Discussion

We present an atypical case of BSLE with clinical, histologic, and immunologic findings, initially suggestive of LABD. Her early presentation, including blisters in annular configurations and IgA deposition at the dermal-epidermal junction on DIF, strongly suggested a diagnosis of LABD (Fig 1, *A*).

Distinguishing BSLE from other autoimmune subepidermal blistering disorders may represent a significant diagnostic challenge owing to multiple overlapping clinical and pathologic features. Classic LABD demonstrates pure or predominant IgA dermal-epidermal junction binding, epidermal (with or without dermal) deposition on SSS, n-serrations on DIF, and IgA autoantibodies against LAD-1, LABD97, BP180 or BP230. IgA-only sublamina densa LABD, the provisional diagnosis in our patient, is now re-classified as IgA epidermolysis bullosa acquisita (IgA EBA) and demonstrates dermal deposition on SSS, u-serrations on DIF, and collagen VII autoantibody targeting, analogous to classic IgG EBA.^1^ In comparison, in BSLE, IgG dermal-epidermal junction binding on DIF is typical, and dermal blinding on SSS, u-serrations on DIF and collagen VII-binding IgG are commonly observed. IgA only DIF staining in BSLE is exceptionally rare with only one case seen out of 20 patients across 2 case series.^2^^,^^3^ Cutaneous exam features may overlap in LABD, IgA EBA, and BSLE.^3^^,^^4^

The main initial clinical feature suggestive of BSLE in this case was the presence of normocytic anemia which prompted serological testing for SLE. Serological testing, along with findings of acute kidney injury, provided major clues to the diagnosis. Thus, in the presence of diagnostic uncertainty concerning an immunobullous disorder with dermal IgA staining, prompt serological evaluation for SLE should be pursued. Other serological features were difficult to interpret; the desmoglein autoantibodies observed were atypical for LABD, EBA, or BSLE, and their clinical significance remains unclear. The negative anticollagen VII IgG ELISA was anticipated given IgA-only deposition on DIF; a clinical test for collagen VII-binding IgA in BSLE was unavailable, but even in classic IgG BSLE collagen VII-binding antibodies are not always detected.^5^

Overlap between LABD and BSLE has been documented previously. In one case involving a middle-aged individual, skin biopsy with SSS staining showed epidermal IgA and positive anti-BP180 C-terminus serology.^6^ However, symptoms were refractory to dapsone and the patient developed lupus nephritis 2 months later, improving only with systemic immunosuppression. Another case involved a child initially diagnosed with LABD.^7^ Two months later, the patient developed fever, polyarthralgia and positive ANA, Smith, and anti-dsDNA titers. In another case, SLE was diagnosed 2 years prior to the development of a blistering disorder with linear IgA only deposition.^8^ In these cases, SSS deposition and serration analyses were not performed. A patient with SLE with linear IgA deposition may therefore have 2 concurrent diagnoses (LABD and SLE) or one diagnosis (BSLE). If considering 2 diagnoses for our patient, the dose of dupilumab could have precipitated SLE, an event previously reported in atopic dermatitis.^9^

Patients with provisional LABD with atypical features or dermal IgA binding should be screened for SLE. Atypical features of LABD include the presence of significant noncutaneous findings such as normocytic anemia and acute kidney injury. Ultimately, understanding the pathomechanisms of BSLE, LABD, and IgA EBA will be crucial to developing precise techniques for the early diagnosis of these conditions and prevention of life-threatening complications.

## Conflicts of interest

None disclosed.
